# A Novel DenseNet Generative Adversarial Network for Heterogenous Low-Light Image Enhancement

**DOI:** 10.3389/fnbot.2021.700011

**Published:** 2021-06-30

**Authors:** Jingsi Zhang, Chengdong Wu, Xiaosheng Yu, Xiaoliang Lei

**Affiliations:** Faculty of Robot Science and Engineering, Northeastern University, Shenyang, China

**Keywords:** DenseNet framework, generative adversarial network, image enhancement, heterogenous low-light image, feature map

## Abstract

With the development of computer vision, high quality images with rich information have great research potential in both daily life and scientific research. However, due to different lighting conditions, surrounding noise and other reasons, the image quality is different, which seriously affects people's discrimination of the information in the image, thus causing unnecessary conflicts and results. Especially in the dark, the images captured by the camera are difficult to identify, and the smart system relies heavily on high-quality input images. The image collected in low-light environment has the characteristic with high noise and color distortion, which makes it difficult to utilize the image and can not fully explore the rich value information of the image. In order to improve the quality of low-light image, this paper proposes a Heterogenous low-light image enhancement method based on DenseNet generative adversarial network. Firstly, the generative network of generative adversarial network is realized by using DenseNet framework. Secondly, the feature map from low light image to normal light image is learned by using the generative adversarial network. Thirdly, the enhancement of low-light image is realized. The experimental results show that, in terms of PSNR, SSIM, NIQE, UQI, NQE and PIQE indexes, compared with the state-of-the-art enhancement algorithms, the values are ideal, the proposed method can improve the image brightness more effectively and reduce the noise of enhanced image.

## Introduction

The imaging process of visible light images is affected by light intensity and environment. The visible light images collected under low light environment have low signal-to-noise ratio, contrast and resolution, which brings more severe challenges to further image processing, such as image recognition and target detection (Shi et al., 2020; Xiaowei et al., 2020; Yin and Li, 2020). Due to the low light environment and limited camera equipment, the image has low brightness, low contrast, high noise, color distortion and other problems, which will not only affect the aesthetics of the image and human visual experience, but also reduce the performance of advanced visual tasks using normal light image. In order to effectively improve the quality of low-light images, scholars have proposed many low-light image enhancement algorithms, which contains three stages: gray scale transformation, retinal cortex theory, and deep neural network (Fukushima, 1980; Gu et al., 2020).

In the early stage, gray scale stretching of the low-brightness area through histogram equalization, gamma correction, and other gray transformation methods (Singh and Kapoor, 2014) can achieve the purpose of improving the brightness of the dark area. However, because the relationship between pixels and their neighbors is not considered, grayscale transformation often leads to the lack of realism in enhanced images.

At present, the common low-light image enhancement methods are mainly divided into four categories: (1) based on the Histogram Equalization method (HE), in this method, the image brightness and contrast can be enhanced by adjusting the histogram distribution. This method is simple and fast, but it often appears color distortion, detail loss and other problems; (2) based on Retinex enhancement method; Cui et al. (2020) proposed that the visual brightness and color perception of human eyes were determined by the reflectivity of the actual object itself and had nothing to do with the intensity of ambient light. According to the Retinex theory, several classical algorithms such as Multi-scale Retinex with Color Restoration (MSRCR) were proposed, which were prone to color distortion (Jiang et al., 2015). The main idea of the MSRCR algorithm is to use Gaussian filter to obtain the light component of low-light image, and then obtain the reflection component through point-by-point operation between pixels as the enhancement result. Tao and Yiquan (2015) used bright-pass filter and logarithmic transformation to balance the brightness and naturalness of the image, so that the enhanced image tended to be natural. Fu et al. (2016) designed a weighted variational model for Simultaneous Reflectance and Illumination Estimation (SRIE), which could effectively deal with the problem of excessive enhancement of dark areas. Guo et al. (2017) proposed Low Light Image Enhancement via Illumination Map Estimation (LIME), which only estimated the illumination component. It mainly used local consistency and structural perception constraints to calculate the reflection component of the image and used it as the output result. Although some scholars added color correction modules, the color distortion problem could not be completely overcome; (3) Based on pseudo-fog image enhancement method. In this method, the inversion image of low illumination image is enhanced by dehazing algorithm. For example, Hu et al. (2020) proposed the enhancement method to achieve better illumination enhancement effect, but block effect and noise were likely to occur when dealing with complex scene enhancement; (4) Based on neural network method. This method uses neural networks to learn the mapping from low light images to normal light images. For example, Chen et al. (2020) proposed to use the convolutional self-encoding network to learn image features from the training set of low-light images. Wang et al. (2019) proposed a multi-branch low-light Enhancement Network (MBLLEN), it learned mapping from the low light image to normal light image. Zhang et al. (2020) proposed a self-supervised illumination enhancement network by combining maximum information entropy and Retinex theory. Ha et al. (2015) designed RetinexNet based on the idea of image decomposition, and adjusted image brightness using decomposition-enhancement architecture. Shi et al. (2019) designed a low light enhancement approach based on RetinexNet. This method could effectively enhance the illumination of low-light images, but the enhanced images were deficient in detail and color.

Deep learning-based low-light image enhancement methods have also been studied. Yang et al. (2016) proposed to enhance low-light images by coupled dictionary learning. Lore et al. (2017) used a deep auto-encoder named Low-Light Net (LLNet) to perform contrast enhancement and denoising. In Shen et al. (2017), deeply root in multi-scale Retinex representation, a feed-forward convolutional neural network with different Gaussian convolution kernels is proposed to learn an end-to-end mapping between dark and bright images. In Wei et al. (2018), Wang et al. proposes a deep Retinex-Net including a Decom-Net for decomposition and an Enhance-Net for illumination adjustment. In Yang et al. (2020) make the attempt in the semi-supervised learning for low-light image enhancement. In this work, a deep recursive band representation is built to connect fully-supervised and un-supervised learning frameworks and integrate their superiorities. The performance of these works rely heavily on the quality of datasets. Due to the lack of a good metric to evaluate various aspects of the overall quality of the enhanced results, e.g., detail preservation, visual naturalness and contrast distribution, their results are not satisfying in some visual aspects.

Generative Adversarial Nets (GAN) was a new generation model proposed by Goodfellow et al. (2014). By observing real data, it learns its potential distribution principle and then generates data that is consistent with the distribution principle.

In order to improve the image quality of low light image, this paper proposes a Heterogenous low-light image enhancement method based on DenseNet generative adversarial network by using the learning ability of generative adversarial network. The main contributions are as follows: The new method uses modified DenseNet structure and deep convolution structure to generate adversarial network, and realizes the use of low light image to generate enhanced image fitting with normal light image. Experimental results show that this new method can effectively improve the brightness and contrast of low light images.

The rest of the paper is organized as follows: The GAN including 3D convolutional neural network is given in section generative adversarial nets (GAN). Next, modified DenseNet architecture is described in section modified DenseNet. Then, the proposed DenseNet GAN and experiment analysis are given in section proposed DenseNet generative adversarial network and section experiments and analysis. This paper is finally concluded in section conclusions.

## Generative Adversarial Nets (GAN)

GAN is composed of generated network (G) and discriminant network (D). The purpose of G is to capture the potential distribution of real data as much as possible. D is a binary classifier, whose purpose is to correctly judge whether the input data comes from the real data or generated data. GAN can be regarded as a mini-max game problem:

(1) $$\begin{array}{l} \underset{G}{\min}\underset{D}{\max}\left(D,G\right)=E_{x\;\sim \;P_{data}\left(x\right)}\left[\log D\left(x\right)\right]\\ \;+E_{z\;\sim \;P_{z}\left(z\right)}\left[\log\left(1-D\left(G\left(x\right)\right)\right)\right] \end{array}$$

Where, E stands for expectation. *x* ~ *P*_*data*_(*x*) is derived from real data. *z* is a random noise subject to a prior distribution *P*_*z*_(*z*) (generally Gaussian distribution, etc.).

Conditional generative adversarial network (CGAN) is proposed to increase the stability of GAN by adding additional condition variable *y* to random noise *z* as a constraint condition. The objective function of CGAN is shown in equation (2):

(2) $$\begin{array}{l} \underset{G}{\min}\underset{D}{\max}V\left(D,G\right)=E_{x\;\sim \;P_{data}\left(x\right)}\left[\log D\left(x|y\right)\right]\\ \;+E_{x\;\sim \;P_{z}\left(x\right)}\left[\log\left(1-D\left(G\left(z|y\right)\right)\right)\right] \end{array}$$

Literature (Fu et al., 2016) proved that GAN could still learn one-to-one mapping from condition variable *y* to real data *x* when random noise *z* is omitted.

## Modified Densenet Architecture

### DenseNet

**Figure 1:**
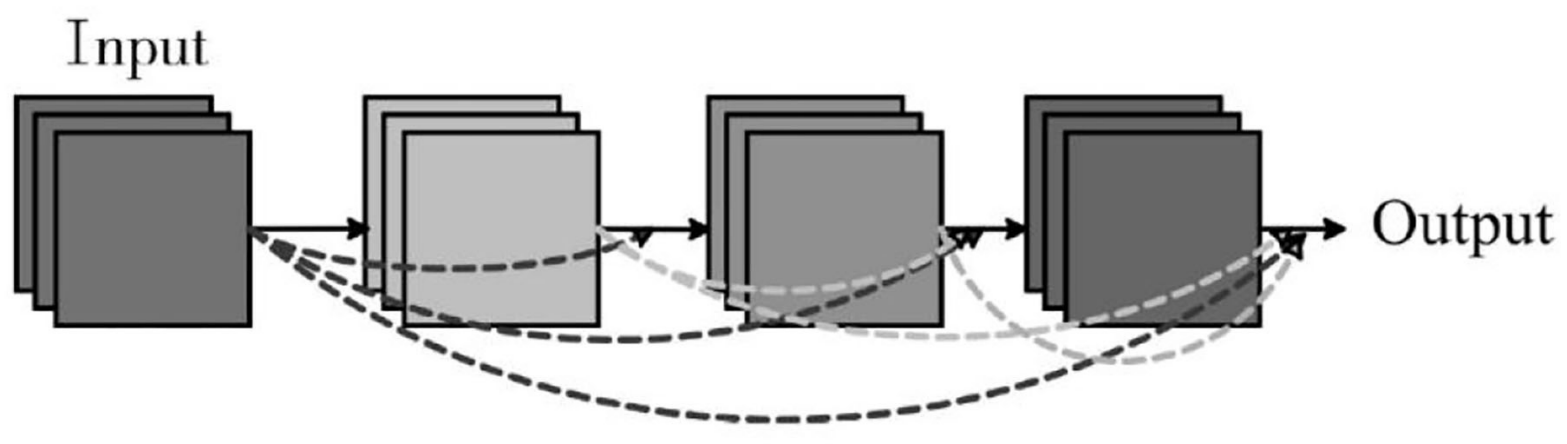
Dense block.

DenseNet is a convolutional neural network with dense connections, which combines the advantages of ResNet and Highway to solve the gradient vanishing problem in deep network (Park et al., 2018). The idea of the DenseNet is to ensure the maximum information transfer between the middle and layers of the network, thus it can directly connect all layers. Figure 1 shows the structure of a Dense block. Block module is the core part of the DenseNet, the main feature is that each layer of the network is not only connected to the next layer (Shoulin et al., 2019), but also directly connected to each layer after this layer. The input of each layer comes from the output of all previous layers. The main reason for the disappearance of gradient is the weak information caused by the transmission of input information and gradient information in the deep network. However, in the Dense block design, each layer is directly connected to input and loss, which can promote the transmission of information, thus reducing the phenomenon of gradient disappearance, and the network can converge better.

In the traditional convolutional neural network, such as AlexNet and VGG, if the network has L layers, then there are L connections. However, in the DenseNet network, the feature graph obtained at the i-th layer is the convolution result of all the previous forward feature graphs after connection, as shown in equation (3).

(3) $$\begin{array}{l} x_{l}=H_{l}\left(\left[x_{0}⊕x_{1}⊕\cdots ⊕x_{l-1}\right]\right) \end{array}$$

Wherein, H represents the convolution operation of this layer, and ⊕ represents the connection operation between feature graphs. In this way, there will be L(L+1)/2 connections in the L layer.

**Figure 2:**
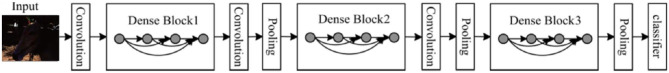
DenseNet structure.

Figure 2 is a basic DenseNet structure diagram, which contains three Dense blocks, in which the 3×3 convolution of each Dense block will be preceded by a 1×1 convolution operation to reduce the number of feature maps. In order to further compress the space, 1×1 convolution layer can be added between two adjacent Dense blocks. Such a network is also called DenseNet-BC network. Compared with the DenseNet with the same depth, it greatly reduces the parameters and also reduces the risk of over-fitting. There are two key parameters in DenseNet that need to be paid attention to. One is the number of feature map output at each layer in the Block *k*, which is called the growth rate. The second is the number of convolution layers *l* in the Block. In this way, the number of output channels of each Dense block is shown in formula (4).

(4) $$\begin{array}{l} S_{out}=S_{0}+k\times l \end{array}$$

Where, *S*_0_ is the channel number input by a single block, and *S*_*out*_ is the channel number output by the Block.

Huang et al. (2017) designed four typical DenseNet-BC networks in the ImageNet object recognition task, they were Densenet-121 (*k* = 32), Densenet-169 (*k* = 32), Densenet-201 (*k* = 32) and Densenet-161 (*k* = 48), respectively. The number of parameters and calculation amount under the same precision are less than that of the ResNet network. As shown in Table 1, the accuracy rate of the four original models in the medical image data set BreakHis is the optimal result under the same experimental environment.

**Table 1:** Accuracy rate with four typical DenseNet-BC networks.

**Model**	**Parameter**	**40X**	**100X**	**200X**	**400X**
Densenet-121 (*k* = 32)	7.05M	85.45	84.05	89.81	84.91
Densenet-169 (*k* = 32)	12.65M	83.98	83.11	86.22	83.04
Densenet-201 (*k* = 32)	18.33M	83.83	84.06	88.19	85.62
Densenet-161 (*k* = 48)	26.69M	79.06	85.49	88.56	89.59

With the increase of DenseNet-BC network parameters, the detection accuracy does not improve correspondingly. The reason is that the more complex network requires more samples to be trained, so as to ensure appropriate parameter update. However, due to the limited samples in BreakHis data set, the network model cannot learn the essential features of breast cancer pathological images well. Therefore, this paper chooses Densenet-121 (*h* = 32) network as the basic network. With high accuracy, the number of parameters of this network is far less than that of the other three network models, which can reduce the memory space, training time and better meet the requirements of real-time image processing.

### Modified DenseNet

In this paper, based on the Densenet-121 model, the application characteristics of low-light image dataset are improved. The main improvement strategies include changing the network structure, enhancing the data set, and transfer learning.

The Densenet-121 (*k* = 32) model is a DenseNet network with a depth of 121 and a growth rate of 32. It is mainly composed of four Block modules, in which the 1 × 1 and 3 × 3 convolution kernel combinations of blocks are 6, 12, 24, and 16 pairs, respectively. The two adjacent blocks are connected by the Translation Layer, which consists of a 1 × 1 convolution Layer and a 2 × 2 pooling layer, further reducing the size of the feature map.

**Figure 3:**
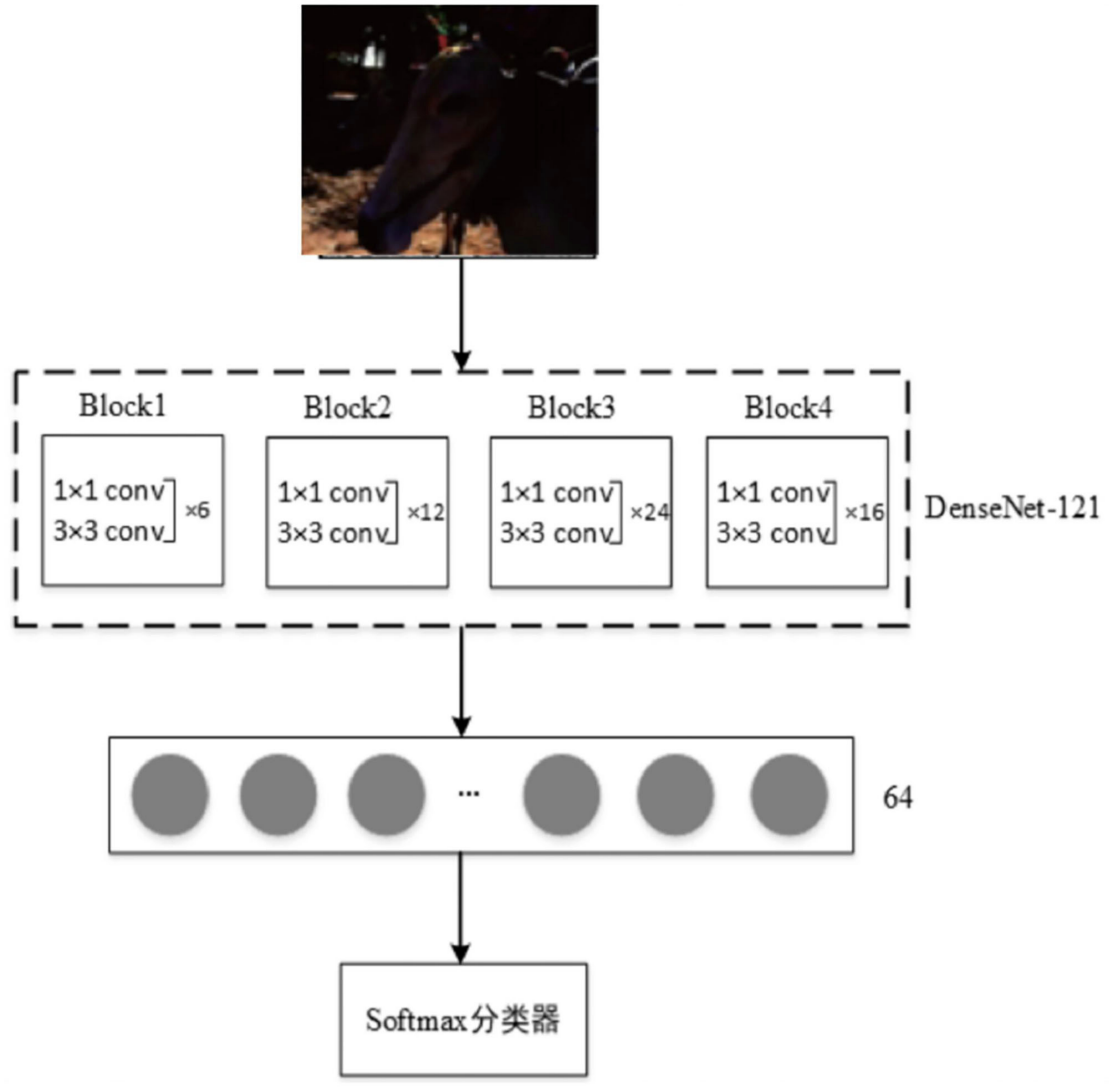
Modified DensNet model.

In this paper, four Block modules are retained on the basis of Densenet-121 network, and the number of neurons in the fully connection layer is changed to 64, while a Softmax classifier is added. The improved network model is shown in Figure 3. The network training is carried out in an end-to-end manner. The four Block modules automatically learn the features of the image from low level to high level, then integrate the essential features through the fully connection layer. Finally, it enhances the image through Softmax classifier.

Softmax classifier is an extension of Logistic regression in multiple classification problems. Firstly, the output of multiple neurons is mapped between (0, 1) through the Softmax activation function to convert the numerical size into probability. Let the input be the array *x*. *X*_*i*_ is the i-th element of the array *x*. The Softmax activation function is calculated as shown in equation (5).

(5) $$\begin{array}{l} f\left(X_{i}\right)=\frac{e^{X_{i}}}{\sum_{j=1}^{k}e^{X_{i}}} \end{array}$$

Where, *k* denotes the number of elements in the array X, but in the proposed model, it represents the number of categories output by the output layer. *f*(*x*) function normalizes the output value of the neural network, then the probability sum of all elements output through *f*(*x*) function is 1. Softmax classifier adopts cross entropy loss function, and the calculation method is shown in equation (6).

(6) $$\begin{array}{l} L_{i}=-\sum y_{i}\log f\left(X_{i}\right) \end{array}$$

In the formula, *y*_*i*_ represents the correct classification label of the image, which is formatted as one-hot coding. In this way, only the probability value of the correct category will be calculated as loss value, and the other values will be zero. Finally, the stochastic gradient descent algorithm is used to minimize the loss function.

## Proposed Densenet Generative Adversarial Network

In the low-light image enhancement problem, low-light image *low* is taken as the condition variable in the network, and the output is the light-enhanced image *G*(*low*). The objective function of low-light enhanced GAN is:

(7) $$\begin{array}{l} \underset{G}{\min}\underset{D}{\max}V\left(D,G\right)=E_{rgb}\left[\log D\left(rgb\right)\right]\\ \;+E_{low}[\log\left(1-D\left(G\left(low\right)\right)\right] \end{array}$$

**Figure 4:**
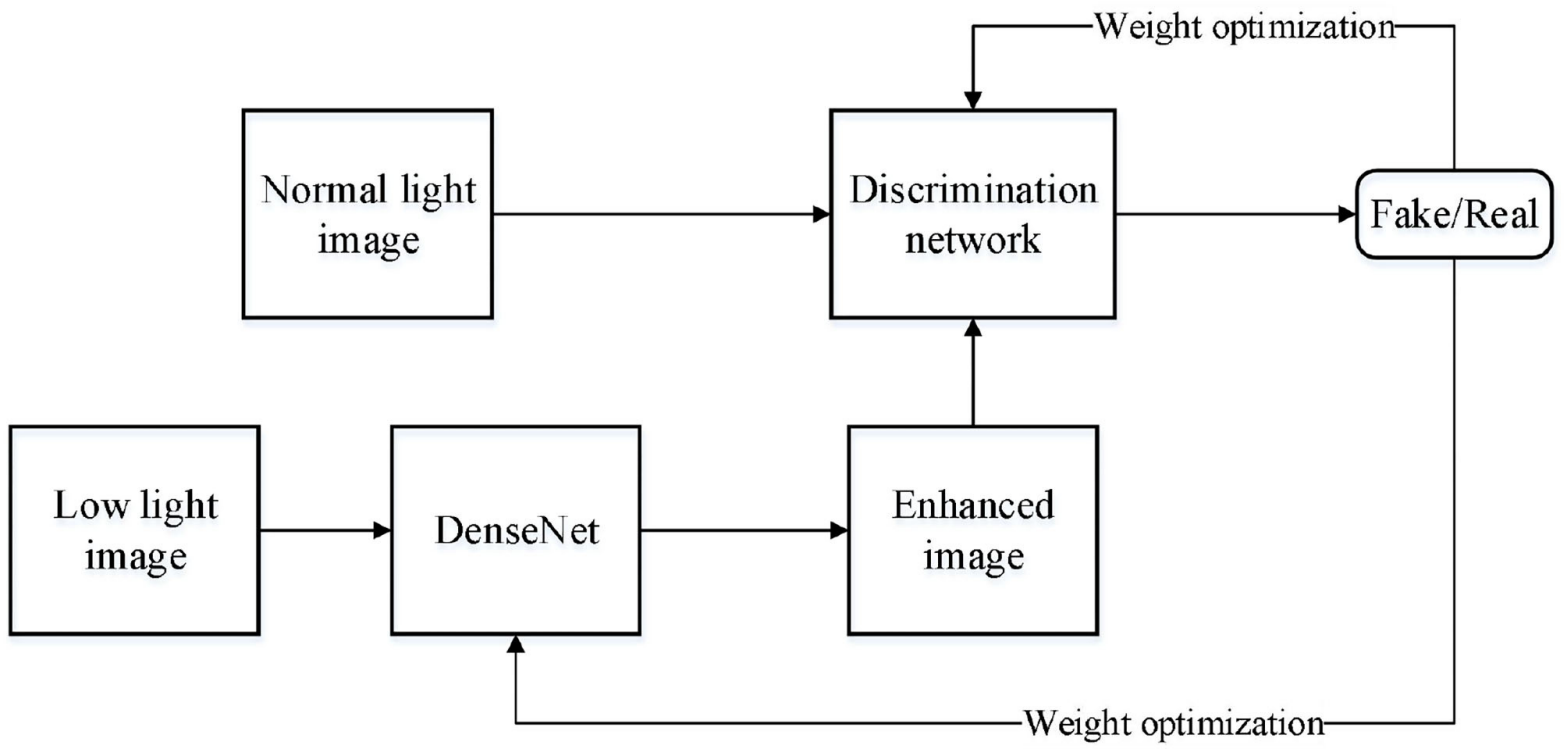
Proposed low-light image enhancement model based on DNGAN.

The proposed model in this paper based on DenseNet GAN (DNGAN) is shown in Figure 4.

The general generative network uses the proposed DensNet as shown in Figure 3. First, the image is divided into blocks, then all the information of the image needs to be compressed into the vector in the full connection layer. Finally, Softmax is used to get the classification result.

In DNGAN network, a non-linear mapping is defined as follows. Firstly, it conducts convolution operation (*) for the input. Then, batch normalization (BN) (Yin et al., 2020) is used to normalize the convolution results. Finally, the activation function is applied to the result. In the generative network, only the output layer uses the Sigmoid activation function, but the rest networks use the ReLU activation function. The discriminant network uses α = 0.2 LeakyRelu activation function. This non-linear mapping structure can increase the ability of the network to extract image features, and the addition of the BN layer can reduce redundant information, accelerate the training speed, and enhance the stability of the network.

Supposing *gF*(*x*) is the non-linear mapping element, *gF*_0_(*x*) = *x*, namely,

(8) $$\begin{array}{l} gF_{j}\left(x\right)=\max\left\{0,BN_{\alpha_{j},\beta_{j}}\left[W_{j}^{*}gF_{j-1}\left(x\right)+b\right]\right\} \end{array}$$

α_*i*_ and β_*i*_ are the reconstruction parameters of BN. If *G*_0_(*x*) = *low* is the input of the generative network. *G*_*i*_(*x*) is the output of coding convolution unit. So,

(9) $$\begin{array}{l} skip\left(x\right)=gF_{i,2}\left[gF_{i,1}\left(G_{i-1}\left(x\right)\right)\right] \end{array}$$

(10) $$\begin{array}{l} G_{i}\left(x\right)=\max pooling\left\{skip\left(x\right)\right\},0<i\leq 3 \end{array}$$

Where *skip*(*x*) represents the jump feature graph, and the bottleneck convolution unit is composed of two non-linear mappings, denoted as *G*_4_(*x*). Before feature extraction, the decoding part of the generative network needs to resize the feature graph and concat it with the jump feature graph, then:

(11) $$\begin{array}{l} up\left(x\right)=concat\left[resize\left(x\right),skip\left(x\right)\right] \end{array}$$

(12) $$\begin{array}{l} G_{i}\left(x\right)=gF_{i,2}\left\{gF_{i,1}\left[up\left(G_{i-1}\left(x\right)\right)\right]\right\},4<i\leq 7 \end{array}$$

Finally, the output layer of generative network is:

(13) $$\begin{array}{l} G\left(low\right)=g\text{\_}out\left(x\right)=G_{8}\left(x\right)=\frac{1}{1+e^{-\left[W_{8}^{*}G_{7}\left(x\right)+b_{8}\right]}} \end{array}$$

## Experiments and Analysis

In this paper, LOL training set (Wei et al., 2018) and synthetic data set are adopted to train the network. The testing set selects LOL, DICM, and MEF data set, which can be downloaded from https://github.com/weichen582/RetinexNet/tree/master/data. In the training process, the Batch Size is 32 and Patch Size is 48 × 48.

The balance coefficient of the network is μ =0.01 and λ =10. Adaptive Moment Estimation (ADAM) is adopted in this paper. The network training and testing experiments are completed on the NVIDIA GTX 2080 GPU, and the implementation code is based on the TensorFlow framework. In order to verify the performance and effect of the proposed algorithm in this paper, the following algorithms are used: SEM (Xie et al., 2020), VBSA (Kim et al., 2020), APM (Feng et al., 2020).

The following objective evaluation indicators are adopted: PSNR, SSIM, NIQE, UQI, NQE, and PIQE (Ou et al., 2021). Bigger SSIM, PSNR, UQI values indicate the better the Image Quality. On the contrary, the higher PIQE, NQE denote the worse image quality. Also the IoU is utilized for evaluating the image enhancement effect. Bigger IoU denotes the better result.

**Figure 5:**
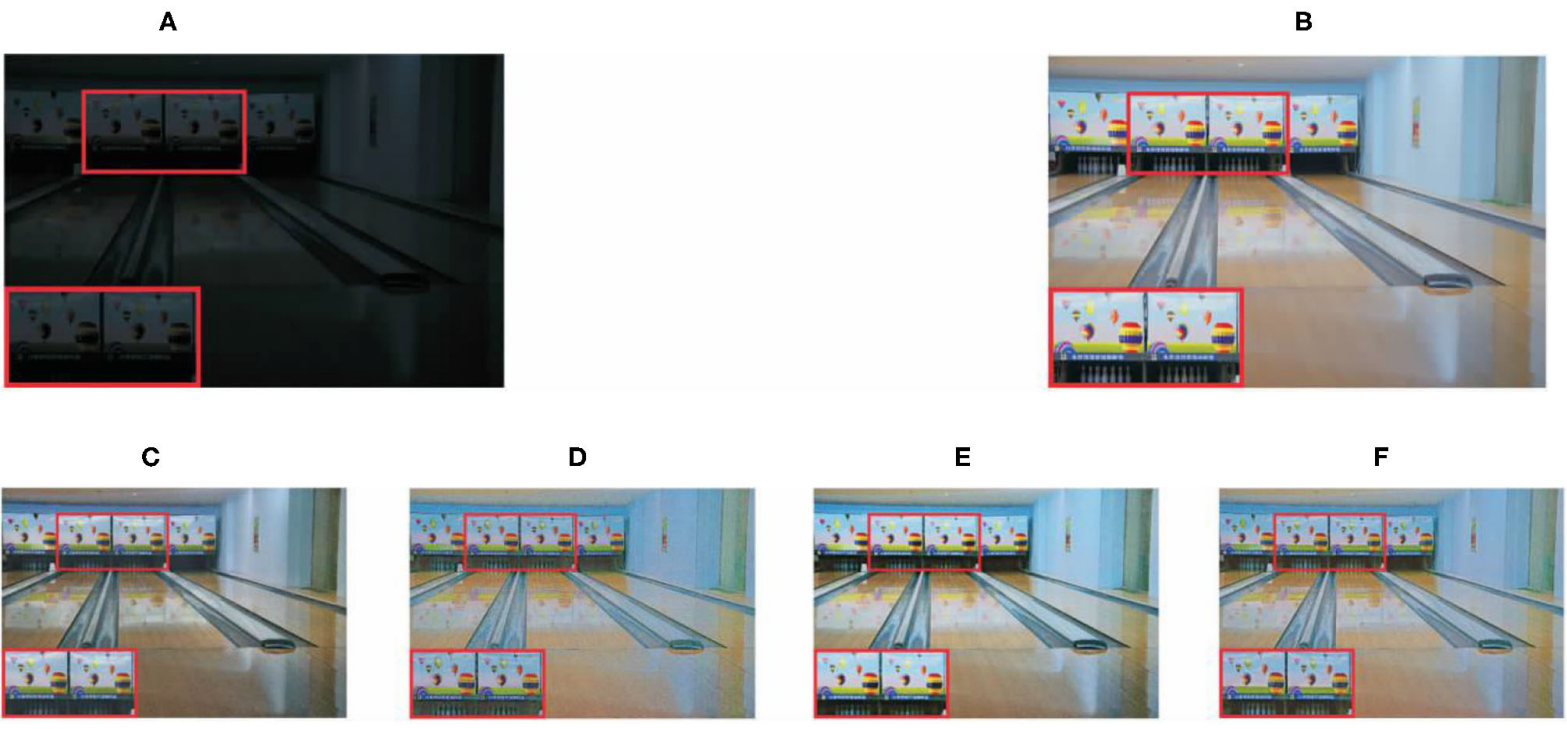
Comparison results with different algorithms on LOL dataset. **(A)** Original image, **(B)** ground truth, **(C)** SEM method, **(D)** VBSA method, **(E)** APM method, and **(F)** proposed method.

**Figure 6:**
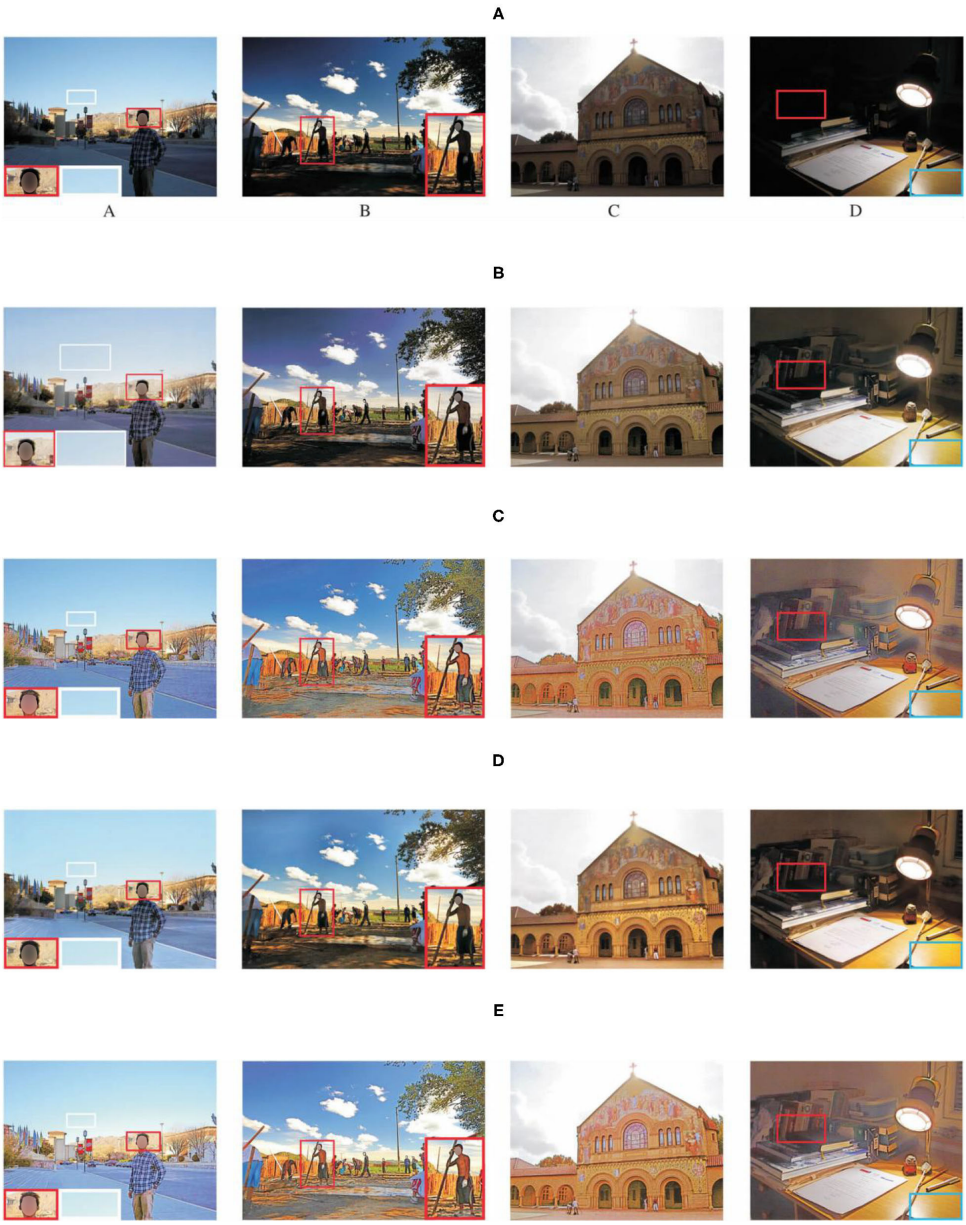
Comparison results with different algorithms on DICM and MEF datasets. **(A)** Input images, **(B)** SEM method, **(C)** VBSA method, **(D)** APM method, and **(E)** proposed method.

Subjective evaluation results are shown in the Figures 5, 6. The red boxes indicate clearly differentiated areas. It shows that the effect of low-light image enhancement is better with the proposed method.

The objective evaluation results of each algorithm on the three data sets are shown in Table 2, where the boldface numbers represent the optimal results and the underlined numbers represent the suboptimal results. For the LOL data set, SSIM can measure the similarity of two images in terms of brightness, contrast and structure, which is highly correlated with the Human Vision System (HVS) and can fully reflect the image quality. As can be seen from Table 2, in terms of SSIM and UQI indexes, the proposed algorithm in this paper achieves the highest value, indicating that the image quality of low-light images is significantly improved. It can be seen from Figures 5, 6 that the algorithm in this paper also improves the expressive force of visual effect. As can be seen from the results of the LOL data set in Table 2, in terms of PSNR index, the proposed algorithm is generally superior to other advanced algorithms. According to the studies in Xie et al. (2020), Kim et al. (2020), and Feng et al. (2020), PSNR index is widely used in image evaluation because it is easy to calculate. However, its calculation is based on error sensitivity, and it often appears inconsistent with human perception system in the evaluation. Therefore, the analysis combined with the subjective effect of the image can better reflect the image quality. Combined with the analysis in Figures 5, 6, the enhanced image with SEM has low saturation and color distortion. The VBSA algorithm makes the image overexposed. Based on DenseNet, the new algorithm significantly reduces the image noise and retains the rich structural information of the image. Compared with other methods, the new algorithm has better visual effect and conforms to the visual perception system of human beings.

**Table 2:** The comparison results on three datasets.

**Method**		**SEM**	**VBSA**	**APM**	**Proposed**
LOL dataset	SSIM	0.512	0.741	0.753	0.822
	UQI	0.536	0.823	0.857	0.896
	PSNR	11.855	15.489	17.963	20.378
	NIQE	8.374	7.924	5.663	4.218
	IoU/%	72.5	77.1	79.8	82.5
DICM dataset	PIQE	16.96	14.78	12.39	10.28
	NIQE	3.886	3.871	3.452	3.323
MEF dataset	PIQE	10.75	8.93	7.41	6.92
	NIQE	3.475	3.716	3.221	3.378

For the LOL data set, compared with other algorithms, the NIQE value obtained in this paper is close to the reference image, indicating that the enhancement result of this algorithm is closer to the reference image. DICM and MEF data sets do not have normal light image as reference. In this paper, only blind image quality assessment index (NIQE, PIQE) is used to evaluate each algorithm, and for the PIQE index, the proposed algorithm obtains the optimal value. For NIQE, the algorithm in this paper achieves better enhancement results compared with DenseNet. In conclusion, although the algorithm in this paper does not achieve the optimal results in all indicators, it still has high advantages. In terms of the SSIM index, the proposed algorithm has good correlation with human visual perception system and the ability of noise suppression. And IoU also is the best with the proposed method.

**Figure 7:**
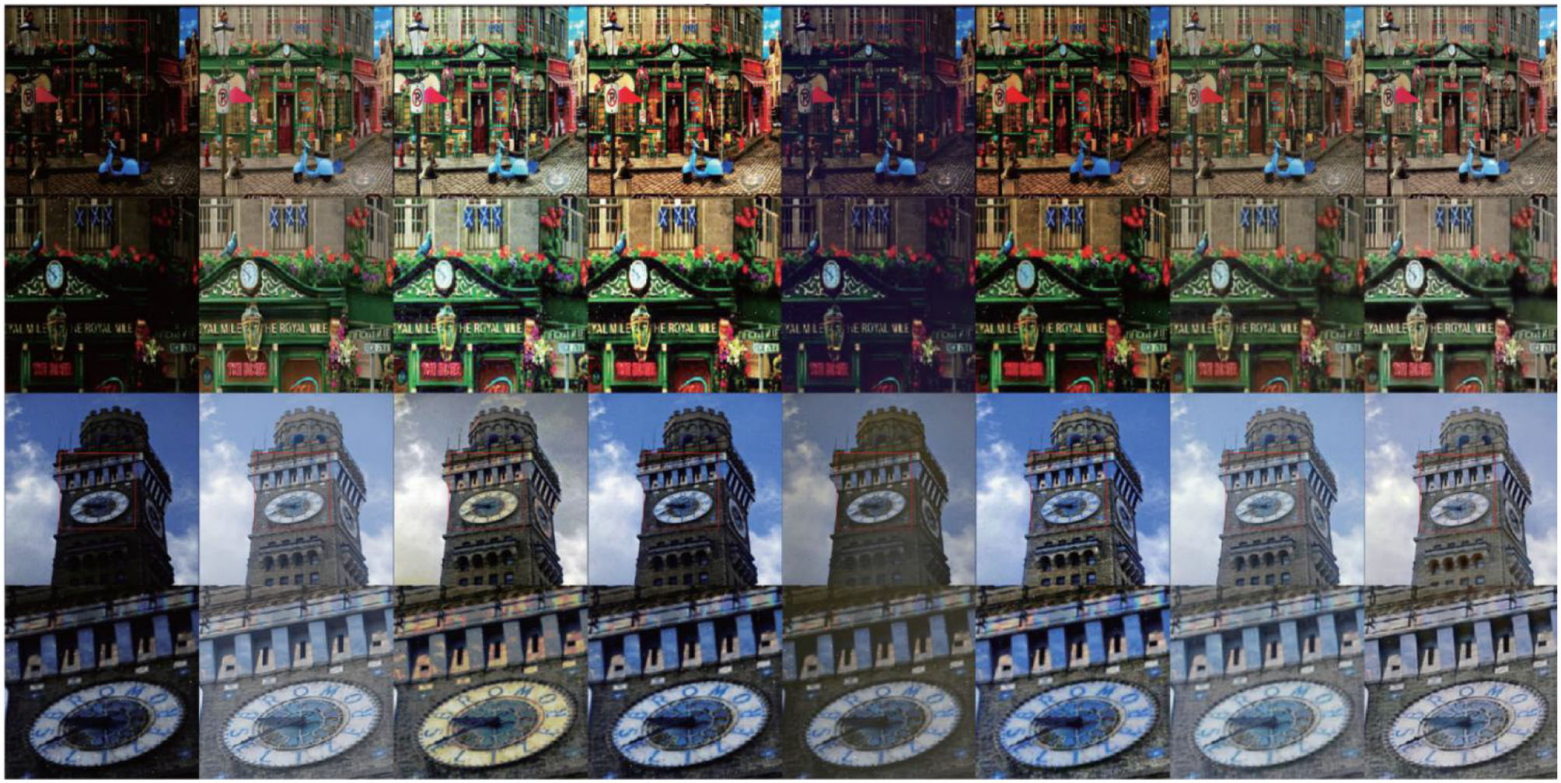
Subjective visual comparison with different algorithms on synthetic low light images. From left to right: original image, ground truth, SEM method, VBSA method, APM method, RetinexDIP (Zhao et al., 2021), Proposed.

We also make comparison experiment on synthetic low-light image. As shown in Figure 7, although SEM and VBSA methods can also improve the illumination problem, the color distortion occurs. They are unable to cope with noise and blur effect. APM results are darker for complex scene images. RetinexDIP method can obviously improve the brightness, but the detail and contrast effect is poor. The proposed model can improve the brightness and contrast better. The rendering is closer to the normal light image. As can be seen from Table 3, the proposed method can obtain better objective evaluation values under the same conditions, that is, the enhanced images are closer to the real images.

**Table 3:** Comparison of objective evaluation indexes with different algorithms on the synthetic low light images.

**Method**	**SEM**	**VBSA**	**APM**	**RetinexDIP**	**Proposed**
SSIM	0.623	0.852	0.864	0.896	0.945
UQI	0.647	0.834	0.868	0.927	0.961
PSNR	11.866	15.583	17.987	21.478	21.489
NIQE	8.255	7.968	5.793	4.375	4.182
IoU/%	69.3	73.1	77.5	80.7	81.6

## Conclusions

Aiming at the problem of low-light image enhancement, a low light image enhancement method based on DenseNet generative adversarial network is proposed in this paper. The retention advantage of DenseNet block structure for image information is utilized to improve the generative network, which improves the performance of the light enhancement model and makes it perform better in color and detail restoration. The characteristics of different loss functions are used to improve the total loss function of the network, so that the network can obtain better light enhancement images with better visual and objective evaluation. The experiment proves that the proposed method is feasible, and compared with some classical light enhancement algorithms, the proposed method in this paper can obtain better light enhancement images. In the future, more deep learning methods will be utilized to perfect the low light image enhancement.

## Data Availability Statement

The original contributions presented in the study are included in the article/supplementary material, further inquiries can be directed to the corresponding authors.

## Author Contributions

JZ and XY: drafting and refining the manuscript. XL and CW: critical reading of the manuscript. All authors have read and approved the manuscript.

## Conflict of Interest

The authors declare that the research was conducted in the absence of any commercial or financial relationships that could be construed as a potential conflict of interest.

## References

[B1] Chen S. Chen Z. Xu X. Yang N. He X. (2020). Nv-Net: efficient infrared image segmentation with convolutional neural networks in the low illumination environment. Infrared Phys. Tech. 105:103184. 10.1016/j.infrared.2019.103184

[B2] Cui Y. Yu Y. Chen Z. Xue B. (2020). Influence of human visual perception and eye tracking motion on the quality of moving image in LCD. IEEE Access 8, 73634–73644. 10.1109/ACCESS.2020.2988766

[B3] Feng X. Li J. Hua Z. (2020). Low-light image enhancement algorithm based on an atmospheric physical model. Multimedia Tools Applicat. 79, 32973–32997. 10.1007/s11042-020-09562-6

[B4] Fu X. Zeng D. Huang Y. Zhang X. Ding X. (2016). “A weighted variational model for simultaneous reflectance and illumination estimation,” in 2016 IEEE Conference on Computer Vision and Pattern Recognition (CVPR). Las Vegas: NVL. 2782–2790. 10.1109/CVPR.2016.304

[B5] Fukushima K. (1980). Neocognitron: a self-organizing neural network model for a mechanism of pattern recognition unaffected by shift in position. Biologic Cybernet. 36, 193–202. 10.1007/BF00344251 7370364

[B6] Goodfellow I. J. Pouget-Abadie J. Mirza M. Xu B. Warde-Farley D. Ozair S. (2014). Generative adversarial networks. Adv. Neural Informat. Proc. Syst. 3, 2672–2680. 10.1145/3422622

[B7] Gu Z. Li F. Fang F. Zhang G. (2020). A novel retinex-based fractional-order variational model for images with severely low light. IEEE Transac. Image Process. 29, 3239–3253. 10.1109/TIP.2019.2958144 31841409

[B8] Guo X. Li Y. Ling H. (2017). LIME: low-light image enhancement via illumination map estimation. IEEE Transact. Image Proc. 26, 982–993. 10.1109/TIP.2016.2639450 28113318

[B9] Ha H. G. Subhashdas S. K. Choi B. S. Ha Y. H. (2015). Hierarchical integrated color matching in a stereoscopic image based on image decomposition. J. Imaging Sci. Tech. 59, 030402.1–030402.7. 10.2352/J.ImagingSci.Technol.2015.59.3.030402

[B10] Hu H. Zhang H. Zhao Z. Li B. Zheng J. (2020). Adaptive single image dehazing using joint local-global illumination adjustment. IEEE Transac. Multimedia 22, 1485–1495. 10.1109/TMM.2019.2944260

[B11] Huang G. Liu Z. Laurens V. Weinberger K. Q. (2017). “Densely connected convolutional networks,” in IEEE Conference on Computer Vision and Pattern Recognition (CVPR) (Honolulu, HI), *Vol. 1*, 2261–2269. 10.1109/CVPR.2017.243

[B12] Jiang B. Woodell G. A. Jobson D. J. (2015). Novel multi-scale retinex with color restoration on graphics processing unit. J. Real-Time Image Proc. 10, 239–253. 10.1007/s11554-014-0399-9

[B13] Kim W. Lee R. Park M. Lee S. H. Choi M. S. (2020). Low-light image enhancement using volume-based subspace analysis. IEEE Access 8, 118370–118379. 10.1109/ACCESS.2020.3005249

[B14] Lore K. G. Akintayo A. Sarkar S. (2017). LLNet: a deep autoencoder approach to natural low-light image enhancement. Pattern Recognit. 61, 650–662. 10.1016/j.patcog.2016.06.008

[B15] Ou J. Xiao H. Jiaxin Y. (2021). Low-light image enhancement algorithm based on improved retinex-Net. Pattern Recog. Artificial Intelligence 34, 77–86. 10.16451/j.cnki.issn1003-6059.202101008

[B16] Park S. Jeong Y. Kim H. S. (2018). Multi-resolution DenseNet based acoustic models for reverberant speech recognition. Phonetics Speech Sci. 10, 33–38. 10.13064/KSSS.2018.10.1.033

[B17] Shen L. Yue Z. Feng F. Chen Q. Liu S. Ma J. (2017). MSRNet: Low-Light Image Enhancement Using Deep Convolutional Network. arXiv:1711.02488. Available online at: http://arxiv.org/abs/~1711.02488

[B18] Shi Y. Wang S. Zhou S. Kamruzzaman M. M. (2020). Study on modeling method of forest tree image recognition based on CCD and Theodolite. IEEE Access 8, 159067–159076. 10.1109/ACCESS.2020.3018180

[B19] Shi Y. Wu X. Zhu M. (2019). Low-Light Image Enhancement Algorithm Based on Retinex and Generative Adversarial Network. arXiv:1906.06027.

[B20] Shoulin Y. Zhang Y. Karim S. (2019). Region search based on hybrid convolutional neural network in optical remote sensing images. Int. J. Distributed Sensor Netw. 15:5. 10.1177/1550147719852036

[B21] Singh K. Kapoor R. (2014). Image enhancement using Exposure based Sub Image Histogram Equalization. Pattern Recog. Lett. 36, 10–14. 10.1016/j.patrec.2013.08.024

[B22] Tao F. Yiquan W. U. (2015). Remote sensing image enhancement based on non-subsampled shearlet transform and parameterized logarithmic image processing model. Acta Geodaet. Cartogr. Sinica 44, 884–892. 10.1117/1.OE.55.10.103104

[B23] Wang L. Fu G. Jiang Z. Ju G. Men A. (2019). “Low-light image enhancement with attention and multi-level feature fusion,” in IEEE International Conference on Multimedia and Expo Workshops (ICMEW) (IEEE), 276–281. 10.1109/ICMEW.2019.00054

[B24] Wei C. Wang W. Yang W. Liu J. (2018). “Deep retinex decomposition for low-light enhancement,” in Proc. Brit. Mach. Vis. Conf (Newcastle). 1–12.

[B25] Xiaowei W. Yin S. Sun K. Li H. Liu J. Karim S. (2020). GKFC-CNN: modified gaussian kernel fuzzy C-means and convolutional neural network for apple segmentation and recognition. J. Appl. Sci. Eng. 23, 555–561. 10.6180/jase.202009_23(3).0020

[B26] Xie J. Bian H. Wu Y. Zhao Y. Shan L. Hao S.. (2020). Semantically-guided low-light image enhancement. Pattern Recogn. Lett. 138, 308–314. 10.1016/j.patrec.2020.07.041

[B27] Yang J. Jiang X. Pan C. Liu C. L. (2016). “Enhancement of low light level images with coupled dictionary learning,” in Proc. 23rd Int. Conf. Pattern Recognit. (ICPR) (Cancun), 751–6. 10.1109/ICPR.2016.7899725

[B28] Yang W. Wang S. Fang Y. Wang Y. Liu J. (2020). “From fidelity to perceptual quality: a semi-supervised approach for low-light image enhancement,” in Proc. IEEE Conf. Comput. Vis. Pattern Recognit (Seattle, WA). 1–10. 10.1109/CVPR42600.2020.00313

[B29] Yin S. Li H. (2020). Hot region selection based on selective search and modified fuzzy C-means in remote sensing images. IEEE J. Select. Top. Appl. Earth Observ. Remote Sens. 13, 5862–5871. 10.1109/JSTARS.2020.3025582

[B30] Yin S. Li H. Teng L. (2020). Airport detection based on improved faster RCNN in large scale remote sensing images. Sens Imaging 21. 10.1007/s11220-020-00314-2

[B31] Zhang Y. Di X. Zhang B. Wang C. (2020). Self-Supervised Image Enhancement Network: Training With Low Light Images Only. arXiv:2002.11300.

[B32] Zhao Z. Xiong B. Wang L. Ou Q. Yu L. Kuang F. (2021). “RetinexDIP: a unified deep framework for low-light image enhancement,” in IEEE Transactions on Circuits and Systems for Video Technology.

